# General existence of locally distinguishable maximally entangled states only with two-way classical communication

**DOI:** 10.1038/srep30181

**Published:** 2016-07-21

**Authors:** Guojing Tian, Xia Wu, Ya Cao, Fei Gao, Qiaoyan Wen

**Affiliations:** 1 State Key Laboratory of Networking and Switching Technology, Beijing University of Posts and Telecommunications, Beijing, 100876, China; 2 State Key Laboratory of Cryptology, P.O. Box 5159, Beijing, 100878, China

## Abstract

It is known that there exist two locally operational settings, local operations with one-way and two-way classical communication. And recently, some sets of maximally entangled states have been built in specific dimensional quantum systems, which can be locally distinguished only with two-way classical communication. In this paper, we show the existence of such sets is general, through constructing such sets in all the remaining quantum systems. Specifically, such sets including *p* or *n* maximally entangled states will be built in the quantum system of (*np* − 1) ⊗ (*np* − 1) with *n* ≥ 3 and *p* being a prime number, which completes the picture that such sets do exist in every possible dimensional quantum system.

Local operations and classical communication (LOCC), as an important operational setting, has been widely considered in the discrimination of quantum states, which has played a significant role in understanding of the relationship between quantum entanglement and quantum nonlocality. On the one hand, quantum entanglement is not necessary for quantum nonlocality^1^,^2^,^3^,^4^,^5^,^6^,^7^,^8^,^9^. Bennett *et al.* firstly discovered a locally indistinguishable 3 ⊗ 3 pure product basis^1^, which has been generalized to higher dimensional quantum systems^2^,^3^,^4^,^5^,^6^, revealing the phenomenon of “nonlocality without entanglement” to a greater extent. As an another interesting finding, there exist orthogonal states whose local indistinguishability is increased with less entanglement^10^. On the other hand, quantum entanglement is not sufficient for quantum nonlocality^11^,^12^,^13^,^14^,^15^,^16^,^17^,^18^,^19^. Any two orthogonal quantum states, entangled or not, can be perfectly distinguished locally^11^. And for maximally entangled states (MESs), Nathanson has proved any three orthogonal MESs in a 3 ⊗ 3 quantum system can be distinguished by LOCC^12^. Then Fan has showed that any *l* mutually orthogonal MESs, which are in canonical forms, are locally distinguishable if *l*(*l* − 1) ≤ 2*p* with *p* being a prime in a *p* ⊗ *p* quantum system^13^, which has been extended to prime power quantum system^14^.

According to the variance of classical communication, there exist two kinds of LOCC settings. We denote local operations and one-way classical communication by “1-LOCC”, and similarly, “2-LOCC” represents local operations and two-way classical communication. Actually, the 2-LOCC is LOCC in common sense, and we will use the expression 2-LOCC to show the difference with 1-LOCC clearly in the following parts. Because of the complexity of 2-LOCC operations, people usually apply either the super sets or the sub sets to study the local discrimination of quantum states. Taking use of the super sets, positive partial transpose (PPT) operations, Yu *et al.* have come up with four locally indistinguishable ququad-ququad MESs^20^, which has been expanded in two power dimensional quantum system^21^,^22^. On the contrary, the researching progresses^11^,^12^,^13^,^14^ have been made mostly based on its subset, 1-LOCC. That is, the local distinguishability of a set is ensured by its 1-LOCC distinguishability. Thus a natural question is whether there exist some quantum states sets or not, which can be distinguished by 2-LOCC but not by 1-LOCC. The existence of such sets, we call 2-LOCC sets, can help to show the difference between 1-LOCC and 2-LOCC, i.e., two-way classical communication has advantage over one-way classical communication in fact. Obviously, the cardinalities of the 2-LOCC sets are small compared to the dimension of their corresponding quantum system, because no *k* > *d* MESs can be locally distinguished^12^,^20^,^21^. In ref. 15, the author has given two triple 2-LOCC sets in the quantum system of dimension 2*m* ⊗ 2*m* and 3r + 2. Lately, we have constructed 2-LOCC sets including four or five MESs in 4*m* ⊗ 4*m* system, and similarly, 3*R* MESs in 2*Rm* ⊗ 2*Rm* system, 4*R* and 5*R* MESs in 4*Rm* ⊗ 4*Rm* system, where *R* = 2^*r*^ with *r* being a positive integer^16^.

However, all the existing results do not cover every possible dimensional quantum system, that is, it is still unknown whether the existence of 2-LOCC sets is general or restricted to specific dimensions. In this paper, we answer this question in positive. We start with presenting a decomposition of all the dimension numbers 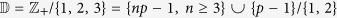, where *p* is a prime number. Combined with the previous result that there exists a 2-LOCC set in even dimensional quantum system, we only need to build 2-LOCC sets in the remaining dimensions {*np* − 1, *n* ≥ 3} because there is no 2-LOCC sets in 3 ⊗ 3 system. The detailed form, *np* − 1, *n* ≥ 3, motivates us to consider using the second 2-LOCC distinguishing protocol in ref. 15 to complete the picture. Before constructing more 2-LOCC sets, we should make a simplification of the referred 2-LOCC distinguishing protocol. Specifically, Bob’s local measurement elements become less, which makes the following construction more efficient. Next, we build *a* + 1 mutually orthogonal MESs in *d* ⊗ *d* quantum system with *d* = *a* + (*a* + 1)*t* base on the simplified 2-LOCC distinguishing protocol. Finally, because of *np* − 1 = *p*(*n* − 1) + (*p* − 1) = *n*(*p* − 1) + (*n* − 1), *n* ≥ 3, we can construct *p* or *n* MESs as 2-LOCC sets easily in the remaining quantum systems, which ensures the general existence of 2-LOCC sets in every possible dimensional quantum system.

## Results

### Notations

Consider a bipartite quantum system *A* ⊗ *B* of the dimension *d* ⊗ *d*, that is, either subsystem *A* or *B* can be regarded as a qudit with *d* levels labeled by 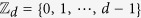. Let 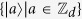 be the computational basis, and the standard bipartite maximally entangled state in this system is expressed as 
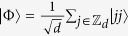
. We can also define the bit flip and phase flip operators to be


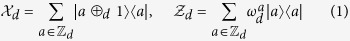


where 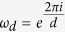 and the subscript denotes the dimension of the corresponding quantum system. When the dimension is 2, the above two operators will become the Pauli matrices *X* and *Z*, respectively. Thus the matrices


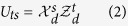


generated by 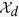 and 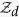 in Eq. (1) have been called generalized Pauli matrices^13^, GPMs for short. Because of the one-to-one relationship between MES and the corresponding unitary operation, we denote the set 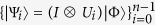 by the corresponding unitary set as 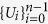, where |Φ〉 is the standard MES.

There has already been a necessary and sufficient condition^15^, which will be employed in the following part repeatedly.

Lemma 1. (*M. Nathanson*^15^) *Given a set of states* 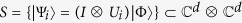, *with* |Φ〉 *the standard maximally entangled state*, *the elements of S can be perfectly distinguished with one-way LOCC if and only if there exists a set of states* 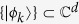 *and a set of positive numbers* {*m*_*k*_} *such that* 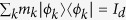 *and*


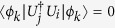


*whenever i* ≠ *j*.

*Equivalently, the elements of S can be perfectly distinguished with one-way LOCC if and only if there exists a d* × *r partial isometry W such that* 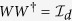 *and such that whenever i* ≠ *j*, *the r* × *r matrix* 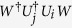 *has every diagonal entry equal to zero*.

### Decomposition of all the dimension numbers

So far, it has already been proved in refs 15 and 16 that there exist 2-LOCC sets in the quantum system of 2*m* ⊗ 2*m*, (3*r* + 2) ⊗ (3*r* + 2), 4*m* ⊗ 4*m*, 2*Rm* ⊗ 2*Rm* and 4*Rm* ⊗ 4*Rm*, where *R* = 2^*r*^ with *r* and *m* being a positive integer. But obviously, the union of {2*m*}, {3*r* + 2}, {4*m*}, {2*Rm*} and {4*Rm*} does not cover all the dimension numbers, or to say all the positive integers greater than one, which motivates us to generalize the result. That is, we want to look for some 2-LOCC set(s) in every-dimension quantum system. Before that, we should be clear about the mathematical structure of all the dimension numbers. Therefore, we will start with a decomposition of all the positive integers, denoted by 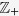, as the following lemma, which may not be the best one but can satisfy our requirements.

Lemma 2. *The set of all dimension numbers*, *denoted by* 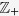, *is equivalent to the union of* {*np* − 1, *n* ≥ 3}, {3} *and* {*p* − 1} *with p being a prime number. That is to say*, 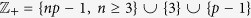.

*Proof*. It is obvious that every number in the union 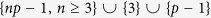 is positive, i.e., 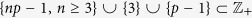. Next, we will explain each positive number in 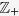 can be expressed as an element in the union.

As the fundamental theorem of arithmetic stated, every integer greater than one either is prime itself or is the product of prime numbers, and that this product is unique, up to the order of the factors. Thus we have 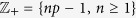 with *p* being a prime, that is, 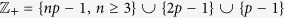. Because the set 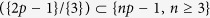 when the latter *p* equals to 2, we can replace {2*p* − 1} by {3} for simplicity. In addition, all the elements in the set {*p* − 1} cannot belong to 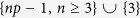, thus we have the final decomposition 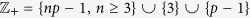, which completes our proof. □

For the dimension 2, any two orthogonal MESs can be discriminated by 1-LOCC^11^, while any three orthogonal MESs cannot be distinguished by 2-LOCC^20^. And for the dimension 3, any three orthogonal MESs are 1-LOCC distinguishable^12^, while any four orthogonal MESs are 2-LOCC indistinguishable^20^. Thus the dimensions of quantum system, denoted by 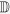, we consider to build 2-LOCC sets should be not smaller than 4, that is,


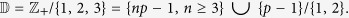


And when the dimension number belongs to {*p* − 1}, which must be even, 2-LOCC sets have been presented in ref. 15. Therefore, our objective becomes to construct 2-LOCC sets in the quantum system of dimension numbers *d* ∈ {*np* − 1, *n* ≥ 3}, where *p* is a prime number. We rewritten the form of “*np* − 1, *n* ≥ 3” to


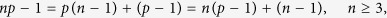


which makes us associate the structure of the second kind of 2-LOCC sets in ref. 15 in the (3*r* + 2) ⊗ (3*r* + 2) quantum system. However, to make full use of that 2-LOCC distinguishing protocol efficiently, we will give it a simpler presentation. That is, a simplified 2-LOCC protocol, we prefer omitting the word “distinguishing” for simplicity, will be discussed as in the following subsection.

### Simplified 2-LOCC protocol

In ref. 15, the author has already proposed two triple MESs sets, which can be distinguished by 2-LOCC but not by 1-LOCC. The 1-LOCC indistinguishability of both sets has been assured by the above sufficient and necessary condition, while the 2-LOCC distinguishability has been obtained by two different protocols. We have successfully employed the first 2-LOCC protocol to construct more 2-LOCC sets in ref. 16. As for the second 2-LOCC protocol to distinguish the three MESs below, actually, it can be simplified further.


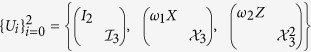


We will present the detailed 2-LOCC distinguishing protocol, which is simpler in Bob’s choice of local measurement. First, Alice does the same measurement as is shown in ref. 15 with 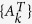, where


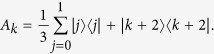


If Alice’s outcome is *k* = 0, without loss of generality, then the original 3 local unitaries *U*_*i*_ will become


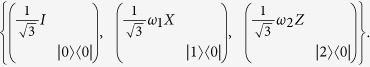


**Figure 1:**
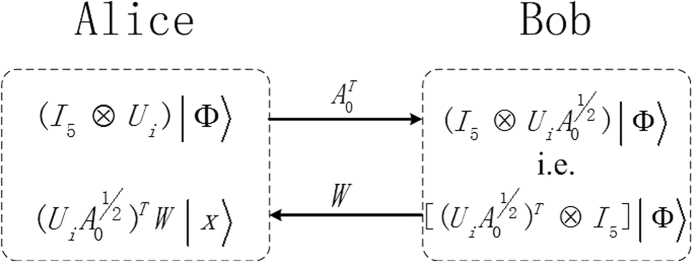
Alice measures with 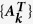 locally and communicates the result 0 to Bob. Then Bob does the measurement {*W*^†^*W*} on his own system and transmits the corresponding result to Alice. At this point, the states in Alice’s part are always mutually orthogonal whatever Bob’s result is, which ensures the 2-LOCC distinguishability.

Next, we will build a simpler *W*, in which each column acts as Bob’s measurement element,


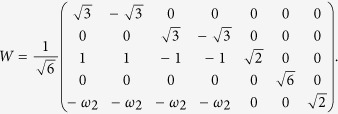


which satisfies the condition that 
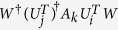
 has diagonal element equal to zero. After Bob transforming this measurement result to Alice, she can finish the local discrimination finally. The whole distinguishing process can be described in the following Fig. 1.

The above 2-LOCC protocol has been much simplified compared to the one given in the ref. 15, because we need only one basis to form the rank-1 measurement operators. That is, we do not need that many measurement elements (*u*, *v*) to distinguish the three MESs using local operations and two-way classical communication.

### Constructing more 2-LOCC sets

Taking use of the above simplified 2-LOCC protocol, we will directly build more 2-LOCC sets of orthogonal MESs in this subsection, which can help us to generalize the existence of 2-LOCC in every possible dimensional quantum system in the next subsection.

Consider the following *a* + 1 mutually orthogonal MESs 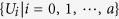 in *d* ⊗ *d* quantum system with *d* = *a* + (*a* + 1)*r* and *r* being an interger, where


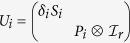


with


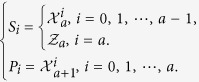


as in Eq. (1), but the dimension is *a*, *a* + 1 respectively. And we should limit the coefficients to satisfy


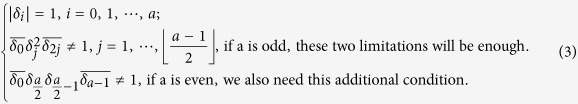


Then we have Theorem as follows.

Theorem 1. *The above defined a* + 1 *mutually orthogonal maximally entangled states* {*U*_*i*_} *are 2-LOCC but not 1*-*LOCC distinguishable*.

*Proof*. We will prove the case of *r* = 1 without loss of generality, because the similar proof method can work for other cases of *r* > 1. To show the 1-LOCC indistinguishability of the *a* + 1 maximally entangled states, we assume there exists a POVM measurement {*M*_*k*_} with every operators *M*_*k*_ all rank-1 to discriminate the above set of states perfectly, which is the necessary and sufficient condition^15^ of 1-LOCC distinguishability. That is, if suppose *M*_*k*_’s diagonal sub-matrices are {*A*_*k*_, *B*_*k*_}, we should have 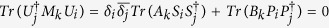, *i* ≠ *j* which implies


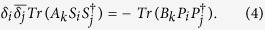


Because the specific forms of *S*_*i*_, 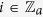 and *P*_*i*_, 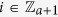, we can write Eq. (4) partly in detail.


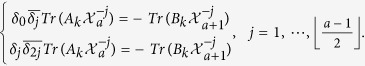


So we have 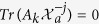 due to the first two eqnarrays in Eq. (3), and further 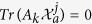, for 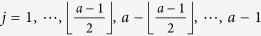, by employing the hermiticity of *A*_*k*_. Similarly, 
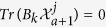
, for 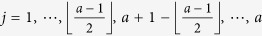. If *a* is odd, re-applying Eq. (4) with 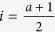, *j* = 0, we can obtain 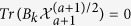. While if *a* is even, we need the third condition in Eq. (3) to obtain 
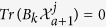
 for 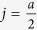, 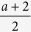. Therefore, 
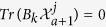
, 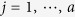.

Reapplying Eq. (4) with 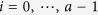; *j* = *a*, there will be 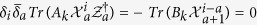, that is, 
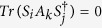
 for 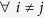 and also 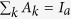. It is a contradiction to the local indistinguishability of *a* +1 maximally entangled states in *a* ⊗ *a* system. So the above set is one-way LOCC indistinguishable.

Then we will prove the 2-LOCC distinguishability through presenting a detailed protocol as follows, which is based on the protocol given in ref. 15 but more generalized and simplified.

*Step 1*. Alice does the measurement with 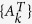, where


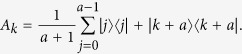


If her outcome is *k*, then the original *a* + 1 local unitaries *U*_*i*_ will become


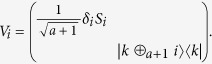


*Step 2*. To discriminate the present *a* + 1 states with certainty, Bob should find out rank-1 measurement operators, one column of *W*, such that


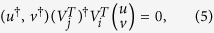


*i*.*e*., 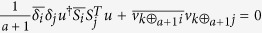. As is referred, the eigenvectors |*e*_*l*_〉 of *S*_*a*_ can make 
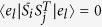
, for *i* < *j* and 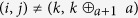. As a result, we can choose 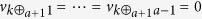 and 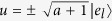. So according to Eq. (5), if we take *v*_*k*_ = *λ*_*l*_, where *λ*_*l*_ is the eigenvalue of *S*_*a*_ corresponding to |*e*_*l*_〉, then 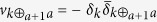. The specific forms of 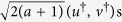 are shown below


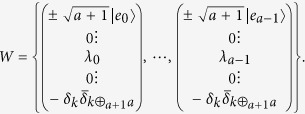


What’s more, we make *u* = 0 and


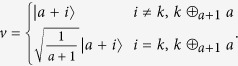


Therefore, the sum of all these rank-1 operators is


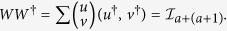


*Step 3*. According to the necessary and sufficient condition in ref. 15, Alice can distinguish the present states based on the measurement outcome of Bob.□

Furthermore, if we choose 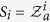, 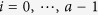 and 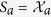, the new set of maximally entangled states also has the property of 2-LOCC but not 1-LOCC distinguishability approaching a similar proving method.

### General existence of 2-LOCC sets

Based on the analysis of all dimension numbers previously, we only need to construct 2-LOCC sets in the quantum system of dimensions *d* ∈ {*np* − 1, *n* ≥ 3} actually. The above “*a* + 1” orthogonal MESs in the quantum system of [(*a* + 1)*r* + *a*] ⊗ [(*a* + 1)*r* + *a*] can help us to explain the existence of 2-LOCC sets in *d* ⊗ *d* quantum system with *d* ∈ {*np* − 1, *n* ≥ 3}, which will prove the fact that 2-LOCC sets are ubiquitous regardless of the dimension of quantum system.

As has already pointed out,


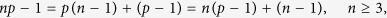


we suppose either *a* = *p* − 1, *r* = *n* − 1 or *a* = *n* − 1, *r* = *p* − 1, and both cases work successfully. That means, there are at least two 2-LOCC sets can be built in the quantum system of (*np* − 1) ⊗ (*np* − 1). If the dimension number plus one, i.e., *np*, have two or more decompositions, then we have more choices to find out 2-LOCC sets. The relationship between the dimension number and the cardinality of a 2-LOCC set in the corresponding quantum system can be shown completely in Table 1. That is, if the dimension is *p* − 1 (*p* ≥ 5), then there exists a 2-LOCC sets including 3 MESs^15^. While if the dimension can be decomposed to *np* − 1 (*n* ≥ 3, *p* ≥ 2), then there exist at least two 2-LOCC sets of *p* or *n* MESs. Until now, we can claim the fact that 2-LOCC sets are general existed regardless of the dimension of quantum system.

Theorem 2. *For every quantum system of dimension number d with d* ≥ 4, *there exist 2-LOCC sets of maximally entangled states*.

Next, to make the existence of 2-LOCC sets more easily to be understood, we will take the dimension *d* = 19 as an example to explain the multiple alternative 2-LOCC sets as well. It should be noted that 19 cannot be expressed in the form of 3*r* + 2, so we must use our method to build 2-LOCC sets. In addition, the parameters which will appear in the following constructions should satisfy the equations in Eq. (3). There exist two decompositions of 19 + 1, i.e., 20 = 4 × 5 = 10 × 2.

When 19 = 4 × 5 − 1, we have

i) 19 = 5(4 − 1) + (5 − 1), and we can construct 5 orthogonal MESs as a 2-LOCC sets as follows


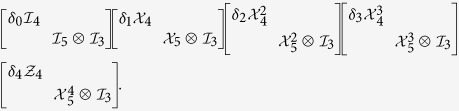


ii) 19 = 4(5 − 1) + (4 − 1), and we can construct 4 orthogonal MESs as a 2-LOCC sets


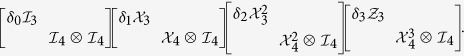


However, when 19 = 10 × 2 − 1, we only have one effective decomposition that 19 = 10(2 − 1) + (10 − 1). The other decomposition 19 = 2(10 − 1) + (2 − 1) is trivial. Thus we can build a 2-LOCC set including 10 orthogonal MESs below


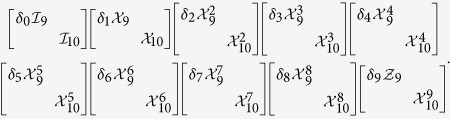


## Discussion

Although there have already existed 2-LOCC sets in many quantum systems, such as 2*m* ⊗ 2*m*, (3*r* + 2) ⊗ (3*r* + 2), 4*m* ⊗ 4*m*, 2*Rm* ⊗ 2*Rm* and 4*Rm* ⊗ 4*Rm*. However, whether this existence is general or not keeps unknown. In this paper, we answer this question in positive. Based on a decomposition of dimension numbers, we become aware that the second 2-LOCC distinguishing protocol can help us to explain the general existence. To employ the 2-LOCC protocol more efficiently, we present a simplified version, which makes us successfully construct *a*+1 MESs as 2-LOCC sets. Therefore, we complete the picture that 2-LOCC sets do exist in every possible dimensional quantum system.

This result leads to a further understanding of the difference between local operations with one-way and two-way classical communication, which is one of the essential topics of quantum information. And we hope the researching progress made in this paper will encourage more researchers to study greater difference between the two locally operational settings.

## Additional Information

**How to cite this article**: Tian, G. *et al.* General existence of locally distinguishable maximally entangled states only with two-way classical communication. *Sci. Rep.* **6**, 30181; doi: 10.1038/srep30181 (2016).

## Figures and Tables

**Table 1:** The relationship between the dimension and number of MESs belonging to 2-LOCC sets.

Dimension	*p* − 1 (*p* ≥ 5)	*np* − 1 (*n* ≥ 3, *p* ≥ 2)
Number of MESs	3	*p* or *n*
